# Comparison of Soft Tissue Changes Produced by Two Different Appliances on Mixed Dentition Children

**DOI:** 10.1155/2021/6612598

**Published:** 2021-03-25

**Authors:** Nashid Fareen, Mohammad Khursheed Alam, Mohd Fadhli Khamis, Norehan Mokhtar

**Affiliations:** ^1^Bangladesh Dental College, Dhaka, Bangladesh; ^2^Orthodontic Division, Preventive Dentistry Department, College of Dentistry, Jouf University, Sakaka, Saudi Arabia; ^3^Forensic Dentistry and Oral Biology Unit, School of Dental Sciences, Universiti Sains Malaysia, Kelantan, Malaysia; ^4^Craniofacial and Biomaterial Sciences Cluster, Advanced Medical and Dental Institute, Universiti Sains Malaysia, Penang, Malaysia

## Abstract

**Objective:**

This study was focused on comparing and analyzing the soft tissue changes induced by Reverse Twin-Block (RTB) and Reverse Pull Face Mask (RPFM) in early and late mixed dentition Malay children having Class III malocclusion.

**Methods:**

This cross-sectional study includes a total sample of 95 Malay children of both early (8-9 years) and late (10-11 years) mixed dentition stages. The comparison was between 49 samples treated by RTB and 46 samples treated by RPFM. Both pre- and posttreatment changes were assessed with Holdaway's analysis using the CASSOS software. In each cephalogram, 71 anatomic landmarks were traced. Descriptive and multiple regression analyses were performed for statistical evaluation.

**Results:**

Statistically significant changes were noticed in soft tissue facial angle, subnasale to H-line, skeletal profile convexity, upper lip strain, H-line angle, lower lip to H-line, and inferior sulcus to H-line measurements. Gender disparity was noticed in upper lip strain. Other significant changes were influenced by the type of appliance. However, the mean differences were minute to notice clinically. Age difference did not have any effect on the treatment changes.

**Conclusions:**

RPFM revealed treatment outcome with more protruded upper lip than RTB.

## 1. Introduction

Treatment of Class III malocclusion in mixed dentition children is considered as a challenging case. As the outcome after growth cessation is unpredictable, orthodontists prefer to treat this particular malocclusion during mixed dentition stage to utilize the effect of the growth spurt as well as reducing the chances of future surgical intervention [1, 2]. The primary goal of orthodontic treatment includes facial esthetics, functional efficiency, and balanced occlusion [1]. Soft tissue change is one of the key factors in improving facial esthetics during orthodontic treatment [3]. Class III malocclusion, either due to retruded maxilla or protruded mandible or by both, exhibits a concave facial profile. Mixed dentition stage is the crucial time to start treatment using orthodontic appliance as sutures are not calcified yet. Some significant changes in dentofacial development take place during this stage [4]. Although soft tissue changes are influenced by the underlying hard tissue changes, the variation in the soft tissue thickness during this stage also contributes greatly [5].

Several studies have successfully used Reverse Twin-Block (RTB) and Reverse Pull Face Mask (RPFM) appliances to treat Class III malocclusion in growing children by modifying the craniofacial growth [2, 6–14]. Whenever there was an evaluation of the clinical effects of these appliances, soft tissue changes were never considered as a prime factor. Holdaway's analysis introduced eleven soft tissue measurements which are renowned for evaluating facial profile harmony [15]. It is well established that considering soft tissue changes during treatment planning brings best possible soft tissue profile [16]. There are only few studies reporting the treatment effect of RTB but none of them emphasized on the soft tissue changes [10–12]. Several studies were found to evaluate the effects of RPFM but still focusing on the dentoskeletal effects [2, 6–10, 13]. Seehra et al. compared both of these appliance's efficacy but the evaluation of soft tissue changes was yet unrevealed.

The ideal time to start the treatment of Class III malocclusion is after the eruption of upper permanent incisors, that is, the beginning of mixed dentition stage [17]. But the debate of whether the treatment should be started immediately or can be delayed until late stage is still unresolved [18]. It is imperative to analyze which appliance brings better efficacy and what should be the ideal age of starting treatment. Hence, the current study is aimed at comparing and analyzing the soft tissue changes produced by RTB and RPFM in early and late mixed dentition Malay children having Class III malocclusion.

## 2. Materials and Methods

### 2.1. Subjects

**Figure 1:**
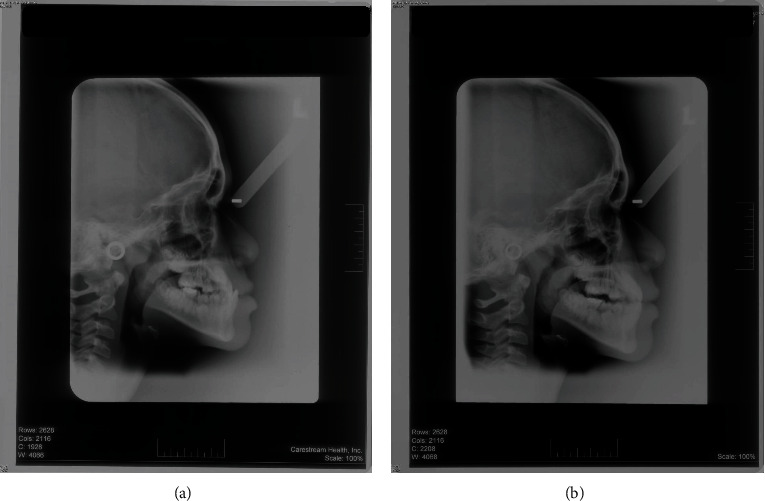
(a) Pre- and (b) posttreatment lateral cephalogram of a patient treated by RPFM.

**Figure 2:**
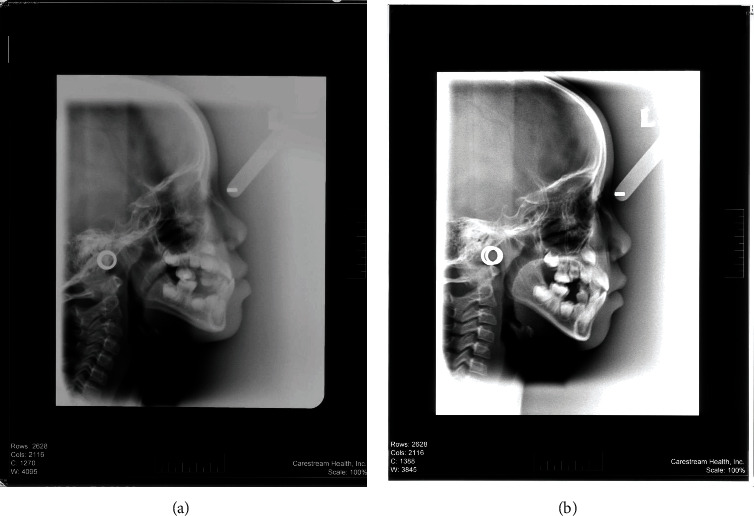
(a) Pre- and (b) posttreatment lateral cephalogram of a patient treated by RTB.

**Figure 3:**
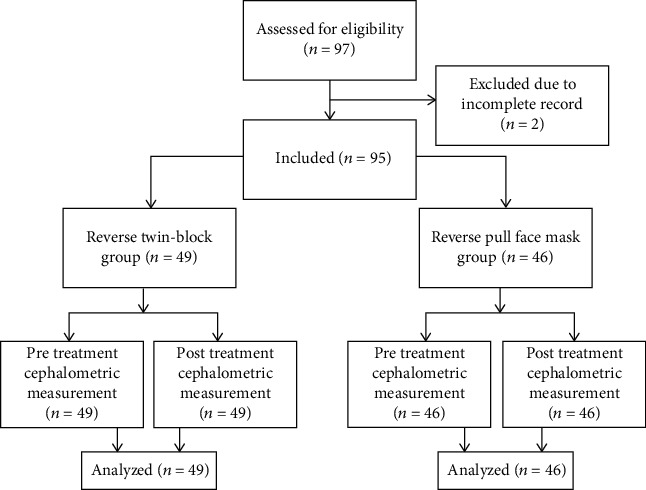
Flow diagram of the study.

This cross-sectional study was approved by the Human Research Ethics Committee (HREC) of Universiti Sains Malaysia (#USM/JEPeM/15070240). The study was carried out by the School of Dental Sciences, Universiti Sains Malaysia, Malaysia. Guideline of Strengthening the Reporting of Observational Studies in Epidemiology (STROBE) was followed [19]. Tabachnik and Fidell formula was used for sample size calculation; *N* > 50 + 8 *m*, where *m* = number of independent variables [20]. Malay children of age 8-9 and 10-11 years having Class III deciduous canine relationship/Class III molar relationship with overjet (-1 to -5 mm) containing clear pre- and posttreatment cephalograms were included in this study. Dental parameter was used for inclusion criteria as the use of cephalogram for sample selection is not ethical. Any patient with craniofacial anomaly, history of facial trauma, and previous orthodontic therapy was excluded. Based on the selection criteria, the samples were extracted from a previously conducted randomized clinical trial [21]. Finally, 49 samples were under RTB and 46 samples were under RPFM. Each group of samples was divided into two age groups, early (8-9 years) and late (10-11 years) mixed dentition groups. The materials used in this study were as follows: (1) pre- and posttreatment lateral cephalograms (Figures 1 and 2) collected retrospectively from the Hospital Universiti Sains Malaysia (HUSM) archive with permission and (2) the Computer-Assisted Simulation System for Orthognathic Surgery (CASSOS 2001, Hong Kong) software for cephalometric tracing and measurements. The flow diagram of the study is presented in Figure 3. Each sample's date of birth and the date of pretreatment cephalogram were used to calculate the age at the beginning of the treatment. This study was located at School of Dental Sciences, Universiti Sains Malaysia.

### 2.2. Appliances

**Figure 4:**
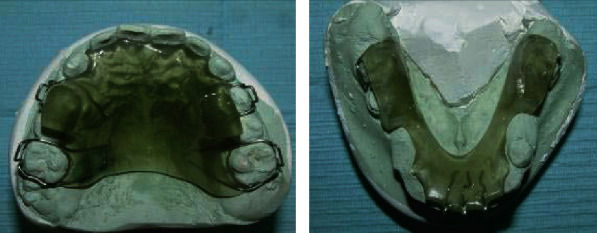
Reverse Twin-Block.

**Figure 5:**
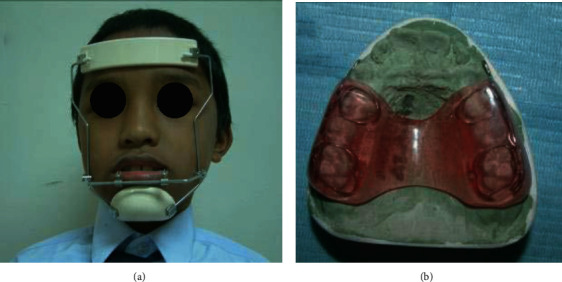
Reverse Pull Face mask (a) extraoral part and (b) intraoral part.

The RTB group received treatment with RTB originally designed by Clark (Figure 4) [22]. Adams clasps on the molars and premolars/deciduous molars in the upper block and in the lower block ball clasps on the incisors were added for retention. The inclined bite planes were angulated at 70° towards the occlusal plane with no labial bow. Full-time wear was encouraged, except during tooth brushing and contact sports. The RPFM group received treatment through Delaire type of facemask with a chin cup, and no expansion screw was attached (Figure 5(a)). A maxillary acrylic splint was used as an intraoral appliance which was banded to the maxillary first permanent molars and first deciduous molars (Figure 5(b)). It covered the maxillary posterior teeth, and the thickness was about two to three millimeters on the occlusal and buccal surface. The extra- and intraoral parts were attached with heavy elastics from the maxillary vestibular hooks (1.0 mm stainless steel wire) to the crossbar of the face mask. The direction of elastic traction was downward and forward. About 400 g of force was applied bilaterally, and 14 hours per day of appliance wear was advised.

**Figure 6:**
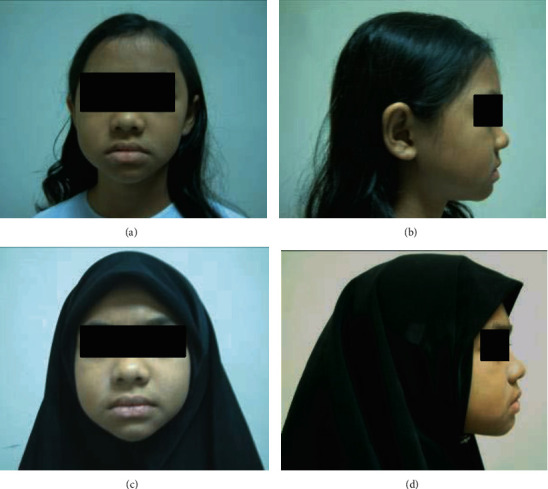
Pretreatment (a) frontal and (b) lateral photograph and posttreatment (c) frontal and (d) lateral photograph of a patient treated by RPFM.

**Figure 7:**
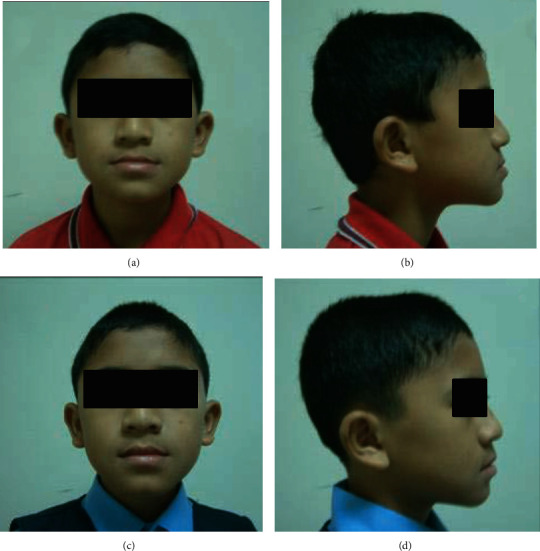
Pretreatment (a) frontal and (b) lateral photograph and posttreatment (c) frontal and (d) lateral photograph of a patient treated by RTB.

Both of the treatment groups received appliance therapy for eight-month period, and then, the treatment change was compared using posttreatment lateral cephalograms. The pre- and posttreatment photographic images of RPFM and RTB appliances are presented in Figures 6 and 7, respectively.

### 2.3. Cephalometric Analysis

**Figure 8:**
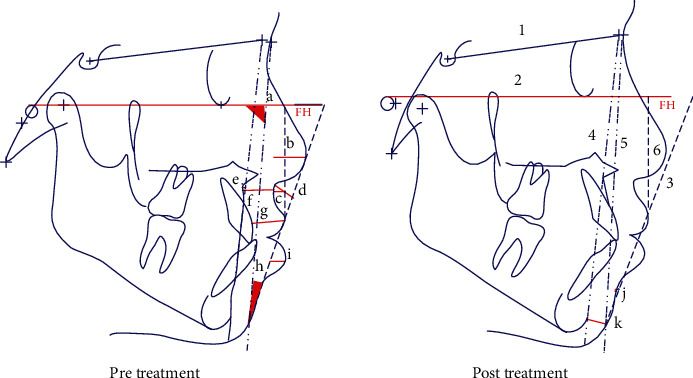
Pre- and posttreatment cephalometric tracing showing reference lines and measurements of Holdaway's analysis. 1, Sella-Nasion (SN) line; 2, Frankfort horizontal (FH) plane; 3, H-line or harmony line; 4, hard tissue facial plane; 5, soft tissue facial line; 6, a line right angle to FH plane downtangent to the vermillion border of upper lip; a, soft-tissue facial angle; b, nose prominence; c, superior sulcus depth; d, subnasale to H-line; e, skeletal profile convexity; f, basic upper lip thickness; g, upper lip strain; h, H-line angle; i, lower lip to H-line; j, inferior sulcus to H-line; k, soft tissue chin thickness.

All cephalograms were taken by the same operator with the same cephalostat. The samples were in centric occlusion and positioned as Frankfort horizontal plane parallel to the floor. All the headfilms were then digitized by VIDAR's DiagnosticPRO® Advantage film digitizer (2010; Herndon, VA, USA) and saved in JPEG format at a resolution, 96 dpi, 1642 × 2086 pixels, and 24 bit grayscale. The cephalograms were traced using the CASSOS software. Images were imported, and 71 landmarks were identified manually using a mouse-driven cursor following which measurements of Holdaway's analysis were generated (Figure 8).

### 2.4. Statistical Analysis

One calibrated researcher traced the lateral cephalograms to avoid interexaminer bias. This researcher was trained and calibrated by a standard examiner. Twenty cephalograms were retraced after two weeks to check the method error using Dahlberg's formula, and it was negligible [23]. All statistical analyses were performed using IBM SPSS Statistics for Windows, Version 22.0 (IBM Corp., Armonk, NY, USA) with significant level set at *P* < .05. The Kolmogorov-Smirnov test proved normal distribution of data in both groups (*P* > .05). Descriptive statistics for means and standard deviation (SD) of each dependent variable were calculated. Multiple regression analysis was performed for evaluation and comparison of the effect of age, sex, and type of appliance on each dependent variable. Dichotomous variables were coded as gender (0 = male and 1 = female), age (0 = early and 1 = late), and type of appliance (0 = RTB and 1 = RPFM).

## 3. Results

### 3.1. Sample

This study focused on comparison between 49 samples treated with RTB and 46 samples treated with RPFM. RTB group had 24 early and 25 late, whereas RPFM group had 20 early and 26 late mixed dentition children.

### 3.2. Descriptive Statistics of Cephalometric Measurements of RPFM Group

Descriptive data of the pretreatment, posttreatment, and treatment changes are presented for early mixed dentition group in Table 1 and for late mixed dentition group in Table 2.

### 3.3. Descriptive Statistics of Cephalometric Measurements of RTB Group

Descriptive data of the pretreatment, posttreatment, and treatment changes are presented for early mixed dentition group in Table 3 and for late mixed dentition group in Table 4.

### 3.4. Regression Analysis of Holdaway Measurements

Multiple linear regression analysis was performed for Holdaway's soft tissue analysis. The data is presented in Table 5. The model assumptions were met; there was no interaction between the independent variables, and no multicollinearity problem was detected. Significant treatment changes were noticed in soft tissue facial angle (*P* = 0.044), subnasale to H-line (*P* = 0.002), skeletal profile convexity (*P* = 0.009), upper lip strain (*P* = 0.012), H-line angle (*P* < 0.001), lower lip to H-line (*P* = 0.013), and inferior sulcus to H-line (*P* = 0.019) measurements. All these changes were affected by the type of appliance except upper lip strain, which showed gender disparity. Patients treated with RPFM had more changes in soft tissue facial angle, subnasale to H-line, skeletal profile convexity, H-line angle, lower lip to H-line, and inferior sulcus to H-line measurements than the patients treated with RTB. Treatment change in upper lip strain was more in boys than in girls. Different age group has not affected significantly on any of the treatment changes.

## 4. Discussion

Analysis of soft tissue changes provides basic information to achieve esthetic harmony, one of the main goals of orthodontic therapy. Facial appearance is mostly dependent on the soft tissue profile [24]. To acquire knowledge about soft tissue morphology along with dentoskeletal analysis, cephalometric soft tissue analysis is essential [25]. It can never be expected that correction of malocclusion only will eventually improve facial esthetics [26]. Soft tissue profile may vary due to many factors, such as race, age, sex, and type of malocclusion [27, 28]. The soft tissue profile is portrayed by chin prominence and thickness; facial profile angle and convexity; nose prominence and nasolabial angle; upper lip length, protrusion, and thickness; lower lip thickness, contour, and relation to the inferior sulcus [3, 24, 25, 27–29]. In our previous studies, craniofacial changes and changes in airway space of these two appliances were compared but detailed comparison of soft tissue changes is focused in this part [30, 31].

The mean and standard deviation of pretreatment, posttreatment, and treatment changes values are presented in Tables 1–4. Significant treatment changes were noticed in seven out of eleven measurements, soft tissue facial angle, subnasale to H-line, skeletal profile convexity, upper lip strain, H-line angle, lower lip to H-line, and soft tissue chin thickness (Table 5). Significant treatment changes were associated with type of appliance except upper lip strain, which showed gender disparity.

An innate relationship persists between facial profile convexity and facial esthetics [32]. Facial profile angle and convexity is established by skeletal profile convexity and H-line angle measurements. An increase in skeletal profile convexity also increases the H-line angle [3]. In this study, children under RPFM group have 0.91 mm more convex skeletal profile and 2.26° larger H-line angle than children under RTB group. This finding suggests an improved soft tissue profile after treatment with RPFM. Soft tissue facial angle and soft tissue chin thickness are associated with chin prominence and thickness [3]. In the current study, soft tissue facial angle in children under RPFM group is 1.35° smaller than in children under RTB group. Soft tissue chin is 0.24 mm thicker in RPFM group than in RTB group. Other studies confirmed that a less concave facial profile is created with decreased soft tissue facial angle or retruded soft tissue pogonion [9, 25, 33]. Therefore, statistically RPFM-treated children have straighter profile than RTB-treated children, but clinically, the difference is too small to notice. Skeletal profile convexity, H-line angle, soft tissue facial angle, and soft tissue chin thickness measurements revealed that both RPFM and RTB produce a harmonious and straighter profile.

A balance in nasal prominence, lip protrusion, and chin prominence maintains facial harmony [5, 34]. No significant difference was noticed in nose prominence after treatment. Isiekwe et al. also found no significant change in nose prominence; Basciftci et al. and Taki et al. found sexual dimorphism but those studies were on adult population [35–37].

Upper lip length, thickness, and protrusion are correlated with superior sulcus depth, subnasale to H-line, basic upper lip thickness, upper lip strain, and H-line angle. Significant changes were observed in subnasale to H-line, upper lip strain, and H-line angle values. Subnasale to H-line indicates upper lip position and thickness. H-line angle denotes upper lip protrusion relating to the facial profile [3, 29]. In the present study, subnasale to H-line and H-line angle values were significantly influenced by type of appliance. Subnasale to H-line value was 0.8 mm more and H-line angle 2.26° larger in RPFM group than in RTB group. These recommend a thicker and harmonious position of the upper lip in RPFM-treated children. More protruded upper lip was noticed in children treated with RPFM due to 2.6° difference of H-line angle. Treatment change in the upper lip strain was significantly affected by the independent variable, gender. Upper lip in boys was 0.94 mm more strained than girls, which indicates girls have a thicker upper lip than boys. Other studies also found gender disparities in lip thickness [38]. However, this small difference is clinically unnoticeable.

In the current study, no significant change was observed in superior sulcus depth, which determines upper lip curl. Other studies also did not notice any significant change [29, 34].

The lower lip position and contour were determined by the lower lip to H-line and inferior sulcus to H-line measurements. Lower lip to H-line value was significantly affected by type of appliance. A more retruded lower lip can be seen in RPFM group as the value was 0.55 mm less in children treated by RPFM than in children treated by RTB. But only 0.55 mm does not make any significant difference clinically. Type of appliance has significant impact on inferior sulcus to H-line measurement, but this value has less importance in indicating soft tissue changes [3].

The results from this study demonstrated a significant association of the appliance type with the majority of the variables. The gender disparity was only noticed in upper lip strain. Although these differences are statistically significant, clinically, they are too small to notice. From clinical point of view, these two appliances produced similar treatment effects on soft tissue changes, except upper lip protrusion. The treatment effect was similar in both early and late mixed dentition stages. If the treatment is started at late mixed dentition stage, we get the benefits of better patient cooperation, less time for treatment follow-up, and moreover better treatment outcome.

With the limitation of using two-dimensional measurements, the use of three-dimensional analysis for future studies with prospective design and long-term follow-up is encouraged.

## 5. Conclusion

- RPFM showed better treatment outcome with more protruded upper lip than RTB
- No significant differences were noticed in the treatment changes between early and late mixed dentition stages
- Male children had more strained upper lip than female children, but the difference was clinically negligible

## Figures and Tables

**Table 1:** Descriptive statistics of early mixed dentition group of RPFM.

Variable	*T*_1_ (*n* = 20)		*T*_2_ (*n* = 20)		*T*_3_ = *T*_2_ − *T*_1_	
	Mean	SD	Mean	SD	Mean	SD
Soft tissue facial angle (°)	90.04	3.91	88.56	4.31	-1.48	3.60
Nose prominence (mm)	10.94	1.41	10.93	1.60	-0.01	0.85
Superior sulcus depth (mm)	3.25	1.78	2.88	1.82	-0.37	0.90
Subnasale to H-line (mm)	7.08	2.64	7.61	2.58	0.54	0.71
Skeletal profile convexity (mm)	0.68	2.76	1.98	2.37	1.31	1.82
Basic upper lip thickness (mm)	12.84	1.23	13.12	1.52	0.28	1.15
Upper lip strain (mm)	15.72	1.74	15.43	2.32	-0.29	1.62
H-line angle (°)	13.96	3.07	15.98	2.91	2.03	2.01
Lower lip to H-line (mm)	3.30	2.00	2.56	2.04	-0.70	1.43
Inferior sulcus to H-line (mm)	1.86	1.45	2.45	1.62	0.59	1.01
Soft tissue chin thickness (mm)	12.35	2.03	12.60	2.95	0.26	2.23

*T*_1_: pretreatment; *T*_2_: posttreatment; *T*_3_: treatment changes; SD: standard deviation.

**Table 2:** Descriptive statistics of late mixed dentition group of RPFM.

Variable	*T*_1_ (*n* = 26)		*T*_2_ (*n* = 26)		*T*_3_ = *T*_2_ − *T*_1_	
	Mean	SD	Mean	SD	Mean	SD
Soft tissue facial angle (°)	91.83	4.71	90.08	4.90	-1.75	3.14
Nose prominence (mm)	12.29	1.98	12.21	1.90	-0.47	0.97
Superior sulcus depth (mm)	3.53	1.97	3.12	1.69	-0.42	0.89
Subnasale to H-line (mm)	6.60	2.84	6.90	2.99	0.31	1.27
Skeletal profile convexity (mm)	0.43	3.19	1.82	3.22	1.38	1.64
Basic upper lip thickness (mm)	13.66	2.35	13.83	2.20	0.17	1.66
Upper lip strain (mm)	16.12	2.00	15.46	1.80	-0.66	2.08
H-line angle (°)	12.47	3.84	14.17	4.56	1.70	2.48
Lower lip to H-line (mm)	3.32	1.92	2.65	2.25	-0.68	1.31
Inferior sulcus to H-line (mm)	2.39	1.75	2.79	2.14	0.40	0.97
Soft tissue chin thickness (mm)	13.47	2.79	13.56	3.26	0.10	1.78

*T*_1_: pretreatment; *T*_2_: posttreatment; *T*_3_: treatment changes; SD: standard deviation.

**Table 3:** Descriptive statistics of early mixed dentition group of RTB.

Variable	*T*_1_ (*n* = 24)		*T*_2_ (*n* = 24)		*T*_3_ = *T*_2_ − *T*_1_	
	Mean	SD	Mean	SD	Mean	SD
Soft tissue facial angle (°)	89.43	4.07	88.82	3.90	-0.61	3.29
Nose prominence (mm)	11.04	1.83	11.22	1.32	0.18	1.39
Superior sulcus depth (mm)	3.22	1.59	2.82	1.53	-0.40	1.26
Subnasale to H-line (mm)	7.26	2.67	6.80	2.65	-0.46	1.28
Skeletal profile convexity (mm)	1.55	2.15	2.21	2.48	0.66	1.57
Basic upper lip thickness (mm)	11.81	2.38	12.03	2.08	0.22	1.35
Upper lip strain (mm)	14.88	3.06	14.35	2.94	-0.53	1.59
H-line angle (°)	14.93	3.56	14.45	3.63	-0.48	2.20
Lower lip to H-line (mm)	3.12	1.41	2.68	1.51	-0.44	0.89
Inferior sulcus to H-line (mm)	2.07	1.48	2.02	1.53	-0.05	1.13
Soft tissue chin thickness (mm)	12.38	2.97	12.13	2.60	-0.25	2.40

*T*_1_: pretreatment; *T*_2_: posttreatment; *T*_3_: treatment changes; SD: standard deviation.

**Table 4:** Descriptive statistics of late mixed dentition group of RTB.

Variable	*T*_1_ (*n* = 25)		*T*_2_ (*n* = 25)		*T*_3_ = *T*_2_ − *T*_1_	
	Mean	SD	Mean	SD	Mean	SD
Soft tissue facial angle (°)	91.00	3.78	91.01	4.17	0.01	2.91
Nose prominence (mm)	11.68	1.60	12.01	1.62	0.33	0.85
Superior sulcus depth (mm)	3.40	1.81	3.14	1.97	-0.26	1.04
Subnasale to H-line (mm)	7.80	3.24	7.48	3.21	-0.32	1.53
Skeletal profile convexity (mm)	1.90	2.37	2.14	2.36	0.24	1.62
Basic upper lip thickness (mm)	13.34	1.63	13.48	1.86	0.15	1.92
Upper lip strain (mm)	15.42	2.09	15.50	2.26	0.07	1.87
H-line angle (°)	15.39	4.97	15.06	4.77	-0.33	1.78
Lower lip to H-line (mm)	3.96	2.21	4.10	2.19	0.14	1.04
Inferior sulcus to H-line (mm)	1.92	1.74	1.96	1.67	0.03	0.88
Soft tissue chin thickness (mm)	13.23	2.34	13.32	2.50	0.08	1.74

*T*_1_: pretreatment; *T*_2_: posttreatment; *T*_3_: treatment changes; SD: standard deviation.

**Table 5:** Results for multiple linear regression of cephalometric variables of Holdaway analysis.

Variable		Coefficients		*t*	*P* value	95% CI for *b*		*R* ^2^
Dependent	Independent	*b*	SE			Lower	Upper	
Soft tissue facial angle (°)	Constant	0.586	1.763	0.333	0.740	-2.916	4.088	0.04
	Age	0.195	0.666	0.293	0.770	-1.127	1.517	
	Sex	0.116	0.667	0.174	0.862	-1.210	1.442	
	Type of appliance	-1.353	0.664	-2.038	0.044^∗^	-2.672	-0.034	

Nose prominence (mm)	Constant	0.820	0.566	1.448	0.151	-0.305	1.945	0.03
	Age	0.039	0.214	0.181	0.857	-0.386	0.463	
	Sex	-0.208	0.214	-0.972	0.334	-0.634	0.217	
	Type of appliance	-0.300	0.213	-1.406	0.163	-0.724	0.124	

Superior sulcus depth (mm)	Constant	-0.285	0.565	-0.505	0.615	-1.408	0.837	0.002
	Age	0.047	0.213	0.219	0.827	-0.377	0.471	
	Sex	-0.026	0.214	-0.121	0.904	-0.451	0.399	
	Type of appliance	-0.074	0.213	-0.346	0.730	-0.496	0.349	

Subnasale to H-line (mm)	Constant	-0.405	0.674	-0.600	0.550	-1.744	0.935	0.13
	Age	-0.046	0.255	-0.182	0.856	-0.552	0.459	
	Sex	-0.462	0.255	-1.809	0.074	-0.969	0.045	
	Type of appliance	0.803	0.254	3.162	0.002^∗∗^	0.299	1.308	

Skeletal profile convexity (mm)	Constant	-1.058	0.893	-1.184	0.239	-2.832	0.717	0.10
	Age	-0.170	0.337	-0.505	0.615	-0.840	0.500	
	Sex	0.553	0.338	1.635	0.105	-0.119	1.225	
	Type of appliance	0.905	0.336	2.688	0.009^∗∗^	0.236	1.573	

Basic upper lip thickness (mm)	Constant	1.185	0.843	1.406	0.163	-0.489	2.858	0.04
	Age	-0.100	0.318	-0.316	0.753	-0.732	0.531	
	Sex	-0.581	0.319	-1.821	0.072	-1.214	0.053	
	Type of appliance	0.050	0.317	0.156	0.876	-0.581	0.680	

Upper lip strain (mm)	Constant	1.331	0.967	1.376	0.172	-0.591	3.252	0.07
	Age	0.113	0.365	0.310	0.757	-0.612	0.839	
	Sex	-0.935	0.366	-2.553	0.012^∗^	-1.662	-0.207	
	Type of appliance	-0.273	0.364	-0.748	0.456	-0.996	0.451	

H-line angle (°)	Constant	-1.455	1.155	-1.259	0.211	-3.750	0.840	0.24
	Age	-0.093	0.436	-0.214	0.831	-0.960	0.773	
	Sex	-0.684	0.437	-1.563	0.121	-1.553	0.185	
	Type of appliance	2.256	0.435	5.183	<0.001^∗∗∗^	1.392	3.121	

Lower lip to H-line (mm)	Constant	0.561	0.637	0.881	0.381	-0.704	1.826	0.09
	Age	0.301	0.240	1.254	0.213	-0.176	0.779	
	Sex	-0.392	0.241	-1.626	0.107	-0.871	0.087	
	Type of appliance	-0.553	0.240	-2.306	0.023^∗^	-1.030	-0.077	

Inferior sulcus to H-line (mm)	Constant	-0.806	0.542	-1.487	0.141	-1.882	0.271	0.07
	Age	-0.040	0.205	-0.193	0.847	-0.446	0.367	
	Sex	0.237	0.205	1.156	0.251	-0.170	0.645	
	Type of appliance	0.490	0.204	2.399	0.019^∗^	0.084	0.895	

Soft tissue chin thickness (mm)	Constant	0.013	1.111	0.012	0.991	-2.195	2.221	0.01
	Age	0.089	0.420	0.213	0.832	-0.744	0.923	
	Sex	-0.301	0.421	-0.716	0.476	-1.137	0.534	
	Type of appliance	0.242	0.419	0.578	0.564	-0.590	1.074	

*b*: regression coefficient; SE: standard error; CI: confidence interval; ^∗^*P* < 0.05; ^∗∗^*P* < 0.01; ^∗∗∗^*P* < 0.001; *n* = 95.

## Data Availability

Data that support the findings of this study are available from the corresponding authors, Alam MK and Khamis MF, upon reasonable request.

## Supplementary Materials

Supplementary Materials STROBE checklist.
